# Supplementary material for: Inverse association between total bilirubin and type 2 diabetes in U.S. South Asian males but not females
Source: PLoS One. 2024 Feb 7;19(2):e0297685. doi: 10.1371/journal.pone.0297685 (PMC10849233; doi:10.1371/journal.pone.0297685)
Supplement: S1 File — (DOCX) [file pone.0297685.s001.docx]

**Supplementary Tables/Figures**

| **Table S1. Baseline Characteristics by Bilirubin Quartile in Males without Evidence of Liver Dysfunction** | | | | | |  |
| --- | --- | --- | --- | --- | --- | --- |
| Exam 1: 442,  Exam 1A: 121 | Overall | Quartile 1 (Male: ≤0.5 mg/dl) | Quartile 2 (Male: 0.6-0.7 mg/dl) | Quartile 3  (Male: 0.8, mg/dl) | Quartile 4  (Male: >0.8 mg/dl) | |
| N | 563 | 169 | 125 | 148 | 121 | |
| **Demographics** |  |  |  |  |  | |
| Age, mean (SD) | 58 (10) | 58 (9) | 58 (11) | 59 (9) | 57 (10) | |
| ≥60 years | 252 (45%) | 74 (44%) | 61 (49%) | 69 (47%) | 48 (40%) | |
| Education |  |  |  |  |  | |
| < Bachelor's Degree | 60 (11%) | 18 (11%) | 15 (12%) | 16 (11%) | 11 (9%) | |
| Bachelor's Degree | 358 (64%) | 105 (62%) | 84 (67%) | 89 (60%) | 80 (66%) | |
| >Bachelor's Degree | 145 (26%) | 46 (27%) | 26 (21%) | 43 (29%) | 30 (25%) | |
| Income Category |  |  |  |  |  | |
| <$40,000 | 80 (14%) | 30 (18%) | 13 (10%) | 18 (12%) | 19 (24%) | |
| $40-75,000 | 81 (14%) | 30 (18%) | 18 (14%) | 20 (14%) | 13 (11%) | |
| $75,000-100,000 | 58 (10%) | 17 (10%) | 13 (10%) | 17 (11%) | 11 (9%) | |
| >$100,000 | 344 (61%) | 92 (54%) | 81 (65%) | 93 (63%) | 78 (64%) | |
| Percent Lived in U.S. |  |  |  |  |  | |
| <40% | 157 (28%) | 51 (30%) | 35 (28%) | 38 (26%) | 33 (27%) | |
| 40-60% | 248 (44%) | 70 (41%) | 60 (48%) | 63 (43%) | 55 (45%) | |
| >60% | 157 (28%) | 48 (28%) | 30 (24%) | 46 (31%) | 33 (27%) | |
| **Sociobehavioral Factors** | |  |  |  |  | |
| Smoking Category |  |  |  |  |  | |
| Never | 401 (71%) | 118 (70%) | 91 (73%) | 105 (71%) | 87 (72%) | |
| Ever smoker | 39 (29%) | 51 (30%) | 34 (27%) | 43 (29%) | 34 (28%) | |
| Alcohol Consumption |  |  |  |  |  | |
| Never Drinker | 135 (36%) | 43 (39%) | 32 (39%) | 37 (36%) | 23 (28%) | |
| Exercise, mean (SD) | 1397 (1389) | 1278 (1223) | 1441 (1302) | 1500 (1530) | 1398 (1519) | |
| ≥600 MET-min/wk (%) | 293 (66%) | 76 (58%) | 73 (70%) | 68 (67%) | 76 (72%) | |
| Dietary Consumption [N=134 missing] |  |  |  |  |  | |
| Total caloric intake (kcal, mean (SD) | 1752 (570) | 1753 (586) | 1746 (588) | 1781 (527) | 1728 (578) | |
| % kcal from fat | 29 (5) | 29 (5) | 29 (6) | 29 (4) | 28 (6) | |
| % kcal from carbohydrates | 56 (6) | 56 (6) | 57 (7) | 56 (5) | 57 (7) | |
| % kcal from protein | 14 (2) | 14 (2) | 15 (2) | 15 (2) | 14 (2) | |
| **Examination Factors** |  |  |  |  |  | |
| BMI (kg/m2) | 25.9 (3.7) | 26.4 (4.2) | 25.7 (3.4) | 26.0 (3.6) | 25.5 (3.1) | |
| <22.9 | 120 (21%) | 34 (20%) | 27 (22%) | 31 (21%) | 28 (23%) | |
| 23-27.4 | 277 (49%) | 78 (46%) | 65 (52%) | 74 (50%) | 60 (50%) | |
| 27.5+ | 165 (29%) | 57 (34%) | 32 (26%) | 43 (29%) | 33 (27%) | |
| Waist circumference | 96.7 (9.3) | 98.0 (10.5) | 95.9 (8.4) | 96.8 (9.0) | 95.6 (8.6) | |
| Systolic BP | 127 (15) | 127 (14) | 126 (13) | 128 (14) | 129 (18) | |
| Diastolic BP | 77 (9) | 76 (10) | 76 (9) | 78 (9) | 78 (10) | |
| **Laboratory/Imaging Factors** | |  |  |  |  | |
| HbA1c % | 6.14 (0.91) | 6.42 (1.12) | 6.06 (0.76) | 6.08 (0.83) | 5.89 (0.71) | |
| HDL-c (mg/dl) | 44.8 (10.7) | 43.4 (10.0) | 44.4 (11.3) | 45.8 (10.8) | 46.0 (11.1) | |
| <40 mg/dl for male | 186 (33%) | 65 (38%) | 48 (38%) | 40 (27%) | 33 (27%) | |
| LDL-c (mg/dl) [N=6 missing] | 108 (33) | 106 (32) | 106 (33) | 107 (34) | 112 (33) | |
| >160 mg/dl (%) | 75 (13%) | 26 (16%) | 12 (9.6%) | 17 (11%) | 20 (17%) | |
| Total Cholesterol (mg/dl) | 180 (38) | 179 (40) | 178 (39) | 178 (37) | 184 (36) | |
| Triglycerides (mg/dl) | 139 (80) | 148 (91) | 142 (62) | 129 (63) | 136 (97) | |
| >150 mg/dl (%) | 191 (34%) | 65 (38%) | 49 (39%) | 37 (25%) | 40 (33%) | |
| Total bilirubin (mg/dl) | 0.70 (0.26) | 0.45 (0.06) | 0.60 (0.0) | 0.74 (0.05) | 1.10 (0.23) | |
| Coronary Artery Calcium (CAC) [N=6 missing] | |  |  |  |  | |
| CAC >0 (%) | 358 (64%) | 116 (69%) | 76 (62%) | 96 (65%) | 70 (59%) | |
| 0 | 199 (36%) | 52 (31%) | 47 (38%) | 51 (35%) | 49 (41%) | |
| 1-400 | 284 (51%) | 94 (56%) | 60 (49%) | 72 (49%) | 58 (49%) | |
| >400 | 74 (13%) | 22 (13%) | 16 (13%) | 24 (16%) | 12 (10%) | |
| Common carotid IMT, mm | 0.91 (0.24) | 0.91 (0.23) | 0.95 (0.31) | 0.91 (0.22) | 0.88 (0.21) | |
| Internal carotid IMT, mm | 1.27 (0.49) | 1.32 (0.51) | 1.32 (0.60) | 1.18 (0.37) | 1.25 (0.44) | |
|  |  |  |  |  |  | |
| High Risk of 10-year ASCVD (>=7.5%) [N=7 missing] | 314 (56%) | 97 (59%) | 70 (56%) | 82 (56%) | 65 (54%) | |
| HOMA-IR Score, median (IQR) | 2.79 (1.87-4.34) | 2.80 (1.98-4.94) | 2.82 (1.77-4.83) | 2.91 (2.02-4.28) | 2.72 (1.72-3.81) | |
| **Comorbidities** |  |  |  |  |  | |
| Hypertension | 338 (60%) | 105 (62%) | 68 (54%) | 91 (61%) | 74 (61%) | |
| Type 2 Diabetes | 163 (29%) | 67 (40%) | 30 (24%) | 42 (28%) | 24 (20%) | |
| Dyslipidemia | 405 (72%) | 130 (77%) | 97 (78%) | 96 (65%) | 82 (68%) | |
| Metabolic Syndrome | 220 (39%) | 72 (43%) | 53 (42%) | 58 (39%) | 37 (31%) | |
| **Medication Use** |  |  |  |  |  | |
| Cholesterol-reducing medication Use | 205 (36%) | 61 (30%) | 49 (39%) | 56 (38%) | 39 (32%) | |
| Statin medication use | 190 (34%) | 56 (33%) | 44 (35%) | 53 (36%) | 37 (31%) | |
| Antihypertensive medication use | 215 (38%) | 69 (41%) | 36 (29%) | 59 (40%) | 51 (42%) | |
| Insulin use | 11 (2.0%) | 4 (2.4%) | 2 (1.6%) | 4 (2.7%) | 1 (0.8%) | |
| Metformin use | 112 (20%) | 47 (28%) | 18 (14%) | 32 (22%) | 15 (12%) | |
| Non-Insulin diabetes medication use | 122 (22%) | 50 (30%) | 22 (18%) | 34 (23%) | 16 (13%) | |
| Format: For continuous variables, values are presented as mean (SD), unless otherwise specified. For categorical variables, values are presented as N (%). IMT = intima-media thickness. | | | | | | |

| **Table S2. Baseline Characteristics by Bilirubin Quartile in Females without Evidence of Liver Dysfunction** | | | | | |  |
| --- | --- | --- | --- | --- | --- | --- |
| Exam 1: 394,  Exam 1A: 136 | Overall | Quartile 1  (≤0.4 mg/dl) | Quartile 2  (0.5 mg/dl) | Quartile 3  (0.6 mg/dl) | Quartile 4  (>0.6 mg/dl) | |
| N | 530 | 194 | 146 | 86 | 104 | |
| **Demographics** |  |  |  |  |  | |
| Age, mean (SD) | 56 (9) | 55 (9) | 57 (9) | 57 (9) | 56 (8) | |
| ≥60 years | 187 (35%) | 61 (31%) | 57 (39%) | 39 (45%) | 30 (29%) | |
| Education |  |  |  |  |  | |
| < Bachelor's Degree | 91 (17%) | 31 (16%) | 24 (16%) | 15 (17%) | 21 (20%) | |
| Bachelor's Degree | 246 (46%) | 100 (52%) | 63 (43%) | 39 (45%) | 44 (42%) | |
| >Bachelor's Degree | 193 (36%) | 63 (32%) | 59 (40%) | 32 (37%) | 39 (38%) | |
| Income Category |  |  |  |  |  | |
| <$40,000 | 94 (18%) | 31 (16%) | 23 (16%) | 21 (24%) | 19 (18%) | |
| $40-75,000 | 66 (12%) | 21 (11%) | 23 (16%) | 9 (10%) | 13 (13%) | |
| $75,000-100,000 | 60 (11%) | 14 (7.2%) | 26 (18%) | 9 (10%) | 11 (11%) | |
| >$100,000 | 310 (58%) | 128 (66%) | 74 (51%) | 47 (55%) | 61 (59%) | |
| Percent Lived in U.S. |  |  |  |  |  | |
| <40% | 160 (30%) | 56 (29%) | 47 (32%) | 19 (22%) | 38 (37%) | |
| 40-60% | 226 (43%) | 90 (46%) | 64 (44%) | 35 (41%) | 37 (36%) | |
| >60% | 143 (27%) | 48 (25%) | 35 (24%) | 32 (37%) | 28 (27%) | |
| **Sociobehavioral Factors** | |  |  |  |  | |
| Smoking Category |  |  |  |  |  | |
| Never | 517 (98%) | 186 (96%) | 142 (97%) | 85 (99%) | 104 (100%) | |
| Ever smoker | 13 (2%) | 8 (4%) | 4 (3%) | 1 (1%) | 0 (0%) | |
| Alcohol Consumption |  |  |  |  |  | |
| Never Drinker | 126 (58%) | 51 (60%) | 26 (50%) | 20 (61%) | 29 (63%) | |
| Exercise, mean (SD) MET-min/wk | 1263 (1311) | 1165 (1201) | 1297 (1160) | 1249 (1274) | 1409 (1691) | |
| ≥600 MET-min/wk (%) | 251 (64%) | 105 (64%) | 69 (68%) | 40 (68%) | 37 (54%) | |
| Dietary Consumption |  |  |  |  |  | |
| Total caloric intake (kcal/day) | 1574 (437) | 1553 (410) | 1595 (460) | 1601 (513) | 1571 (402) | |
| % kcal from fat | 29.8 (4.7) | 29.9 (4.6) | 29.0 (5.3) | 29.4 (4.6) | 30.4 (4.7) | |
| % kcal from carbohydrates | 56.2 (5.7) | 56.1 (5.2) | 56.7 (6.3) | 56.0 (5.3) | 55.8 (6.3) | |
| % kcal from protein | 15.0 (2.2) | 15.0 (2.2) | 14.8 (2.2) | 15.5 (2.1) | 14.9 (2.2) | |
| **Examination Factors** |  |  |  |  |  | |
| BMI (kg/m2) | 26.5 (4.4) | 26.5 (4.1) | 26.6 (4.7) | 26.2 (4.7) | 26.5 (4.2) | |
| <22.9 | 113 (21%) | 33 (17%) | 32 (22%) | 23 (27%) | 25 (24%) | |
| 23-27.4 | 225 (42%) | 91 (47%) | 59 (40%) | 32 (37%) | 43 (41%) | |
| 27.5+ | 192 (36%) | 70 (36%) | 55 (38%) | 31 (36%) | 36 (35%) | |
| Waist circumference | 90.5 (10.3) | 90.1 (9.4) | 90.9 (10.5) | 89.7 (10.9) | 91.2 (11.3) | |
| Systolic BP | 124 (17) | 124 (16) | 124 (17) | 124 (18) | 124 (19) | |
| Diastolic BP | 71 (10) | 70 (10) | 72 (10) | 71 (10) | 72 (10) | |
| **Laboratory/Imaging Factors** | |  |  |  |  | |
| HbA1c % | 5.96 (0.80) | 5.99 (0.75) | 6.04 (0.81) | 5.88 (1.02) | 5.85 (0.66) | |
| HDL-c (mg/dl) | 56.2 (13.9) | 54.9 (14.4) | 56.2 (12.7) | 57.3 (14.7) | 57.4 (14.1) | |
| <40 mg/dl for male | 188 (35%) | 80 (41%) | 49 (34%) | 28 (33%) | 31 (30%) | |
| LDL-c (mg/dl) | 114 (32) | 114 (30) | 117 (34) | 115 (33) | 108 (31) | |
| >160 mg/dl (%) | 65 (12%) | 20 (10%) | 26 (18%) | 12 (14%) | 7 (6.7%) | |
| Total Cholesterol (mg/dl) | 194 (36) | 194 (34) | 198 (40) | 195 (37) | 188 (35) | |
| Triglycerides (mg/dl) | 121 (53) | 125 (56) | 123 (54) | 116 (47) | 116 (48) | |
| >150 mg/dl (%) | 124 (23%) | 52 (27%) | 39 (27%) | 14 (16%) | 19 (18%) | |
| Total bilirubin (mg/dl) | 0.54 (0.20) | 0.37 (0.05) | 0.50 (0.0) | 0.60 (0.0) | 0.85 (0.21) | |
| Coronary Artery Calcium (CAC) [N=6 missing] | |  |  |  |  | |
| CAC >0 (%) | 156 (30%) | 58 (30%) | 37 (26%) | 31 (36%) | 30 (29%) | |
| 0 | 372 (70%) | 136 (70%) | 107 (74%) | 55 (64%) | 74 (71%) | |
| 1-400 | 137 (26%) | 54 (28%) | 29 (20%) | 28 (33%) | 26 (25%) | |
| >400 | 19 (3.6%) | 4 (2.1%) | 8 (5.6%) | 3 (3.5%) | 4 (3.9%) | |
| Common carotid IMT, mm | 0.84 (0.20) | 0.82 (0.17) | 0.87 (0.24) | 0.85 (0.20) | 0.82 (0.21) | |
| Internal carotid IMT, mm | 1.14 (0.40) | 1.13 (0.31) | 1.17 (0.48) | 1.24 (0.52) | 1.07 (0.34) | |
| High Framingham Score Risk (>20% or DM) [N=2 missing] | 85 (16%) | 29 (15%) | 29 (20%) | 12 (14%) | 15 (15%) | |
| High Risk of 10-year ASCVD (>=7.5%) [N=2 missing] | 96 (18%) | 32 (16%) | 30 (21%) | 18 (21%) | 16 (16%) | |
| HOMA-IR Score, median (IQR) | 2.25 (1.48-3.36) | 2.30 (1.57-3.49) | 2.28 (1.52-3.33) | 1.82 (1.19-3.24) | 1.79 (1.21-2.98) | |
| **Comorbidities** |  |  |  |  |  | |
| Hypertension | 244 (46%) | 94 (48%) | 61 (42%) | 42 (49%) | 47 (45%) | |
| Type 2 Diabetes | 94 (18%) | 34 (18%) | 33 (23%) | 11 (13%) | 16 (15%) | |
| Dyslipidemia | 326 (62%) | 126 (65%) | 93 (64%) | 50 (58%) | 57 (55%) | |
| Metabolic Syndrome | 195 (37%) | 78 (40%) | 57 (39%) | 27 (31%) | 33 (32%) | |
| **Medication Use** |  |  |  |  |  | |
| Cholesterol-reducing medication Use | 136 (26%) | 52 (27%) | 35 (24%) | 19 (22%) | 30 (29%) | |
| Statin medication use | 128 (24%) | 45 (23%) | 35 (24%) | 18 (21%) | 30 (29%) | |
| Antihypertensive medication use | 149 (28%) | 58 (30%) | 39 (27%) | 25 (29%) | 27 (26%) | |
| Insulin use | 9 (1.7%) | 4 (2.1%) | 3 (2.1%) | 1 (1.2%) | 1 (1.0%) | |
| Metformin use | 63 (12%) | 19 (10%) | 23 (16%) | 10 (12%) | 11 (11%) | |
| Non-Insulin diabetes medication use | 72 (14%) | 25 (13%) | 24 (16%) | 11 (13%) | 12 (11%) | |

| **Supplementary Table S3.** Association between Total Bilirubin Quantiles and Type 2 Diabetes in Participants without Liver Dysfunction | | | | | | | |
| --- | --- | --- | --- | --- | --- | --- | --- |
|  | Men | | | Women | | | |
|  | N | Unadjusted | Adjusted | N | Unadjusted | Adjusted |  |
| Quartile 1 | 67/169 | 2.66 (1.54, 4.57)* | 3.05 (1.66, 5.58)* | 34/194 | 1.17 (0.61, 2.24) | 1.13 (0.53, 2.41) |  |
| Quartile 2 | 30/125 | 1.28 (0.70, 2.34) | 1.62 (0.83, 3.18) | 33/146 | 1.61 (0.83, 3.10) | 1.39 (0.64, 3.02) |  |
| Quartile 3 | 42/148 | 1.60 (0.90, 2.84) | 1.86 (1.00, 3.49) | 11/86 | 0.81 (0.35, 1.85) | 0.62 (0.24, 1.62) |  |
| Quartile 4 | 24/121 | 1.00 (REF) | 1.00 (REF) | 16/104 | 1.00 (REF) | 1.00 (REF) |  |
|  |  |  |  |  |  |  |  |
| Tertile 1 | 67/169 | 2.12 (1.34, 3.37) | 2.32 (1.38, 3.90) | 9/54 | 1.21 (0.53, 2.75) | 1.34 (0.52, 3.41) |  |
| Tertile 2 | 53/212 | 1.08 (0.68, 1.71) | 1.26 (0.75, 2.09) | 58/286 | 1.54 (0.93, 2.53) | 1.59 (0.89, 2.83) |  |
| Tertile 3 | 43/182 | 1.00 (REF) | 1.00 (REF) | 27/190 | 1.00 (REF) | 1.00 (REF) |  |
| Format: N: # of diabetes / total # of subjects. Odds Ratio [95% Confidence Interval]. *p-value of parameter estimate is <0.004 (Bonferroni corrected p-value threshold for significance)  Quartiles: Men: <0.6, 0.6, 0.7-0.8, >0.8; Women: <0.5, 0.5, 0.6, >0.6 mg/dl  Tertiles defined as follows: Male: <0.6, 0.6-0.7, >0.7; Female: <0.4, 0.4-0.5, >0.5 mg/dl.  1. Odds ratios adjusted for age, BMI, education level, household income quartile, percent of life living in the U.S., exercise (in MET-min/week), alcohol consumption (yes/no), metabolic syndrome criteria (except for outcome of interest: HbA1c%, waist circumference, HDL, triglycerides, systolic BP), LDL, CT-based fatty liver, and smoking status (only in males).  Diabetes defined as self-reported diagnosis, fasting plasma glucose ≥126 mg/dl, HbA1c % ≥6.5%, or anti-diabetes medication use. | | | | | | | |

| **Supplementary Table S4.** Odds of Cardiovascular Risk Factors in the Lowest Bilirubin vs. Highest Bilirubin Quartile Among Participants without Liver Dysfunction | | | | |
| --- | --- | --- | --- | --- |
|  | Men | | Women | |
|  | Unadjusted | Adjusted | Unadjusted | Adjusted |
| Type 2 Diabetes | 2.66 (1.54, 4.57) | 3.05 (1.66, 5.58) | 1.17 (0.61, 2.24) | 1.13 (0.53, 2.41) |
| Hypertension | 1.04 (0.65, 1.68) | 0.64 (0.37, 1.09) | 1.14 (0.71, 1.84) | 1.28 (0.76, 2.17) |
| Dyslipidemia | 1.59 (0.94, 2.67) | 1.61 (0.96, 2.70) | 1.53 (0.94, 2.48) | 1.44 (0.86, 2.38) |
| Low HDL | 1.59 (0.94, 2.67) | 1.45 (0.85, 2.49) | 1.53 (0.94, 2.48) | 1.44 (0.84, 2.47) |
| Triglyceridemia | 1.27 (0.78, 2.07) | 1.09 (0.65, 1.83) | 1.64 (0.91, 2.96) | 1.35 (0.72, 2.56) |
| High LDL | 0.94 (0.50, 1.78) | 0.68 (0.34, 1.36) | 1.59 (0.65, 3.90) | 1.68 (0.64, 4.38) |
| Metabolic Syndrome | 1.69 (1.03, 2.76) | 1.35 (0.80, 2.27) | 1.45 (0.88, 2.39) | 1.56 (0.90, 2.70) |
| Obesity | 1.36 (0.81, 2.26) | 1.42 (0.82, 2.45) | 1.07 (0.65, 1.76) | 1.02 (0.60, 1.72) |
| Presence of CAC | 1.56 (0.96, 2.55) | 1.59 (0.88, 2.87) | 1.05 (0.62, 1.78) | 1.05 (0.55, 2.01) |
| High distal common carotid IMT* | 1.19 (0.67, 2.13) | 1.25 (0.64, 2.45) | 1.03 (0.45, 2.36) | 0.91 (0.35, 2.36) |
| High internal carotid IMT* | 1.12 (0.60, 2.06) | 1.13 (0.54, 2.34) | 1.70 (0.66, 4.38) | 1.25 (0.64, 2.45) |
| ASCVD Risk Score ≥7.5% | 0.85 (0.52, 1.37) | 1.09 (0.66, 1.81) | 0.97 (0.50, 1.88) | 1.10 (0.55, 2.22) |
| Format: Odds Ratio [95% Confidence Interval]. IMT=intima-media thickness. CAC=coronary artery calcium. *p-value of parameter estimate is <0.004 (Bonferroni corrected p-value threshold for significance)  1. Odds ratios adjusted for age, BMI category, education level, household income quartile, percent of life living in the U.S., metabolic syndrome criteria (except for outcome of interest), and smoking status (only in males). | | | | |

| **Table S5.** Odds of Cardiovascular Risk Factors as Continuous Covariates in the Lowest Bilirubin vs. Highest Bilirubin Quartile | | | | | |
| --- | --- | --- | --- | --- | --- |
|  | Men | | Women | | |
|  | Adjusted regression coefficient (95% CI)^1^ | P-value | Adjusted regression coefficient (95% CI)^1^ | P-value |  |
| HbA1c% | 0.51 (0.31, 0.71) | <0.0001 | 0.12 (-0.059, 0.31) | 0.19 |  |
| Systolic BP | -4.32 (-7.57, -1.07) | 0.0093 | 0.14 (-3.51, 3.77) | 0.94 |  |
| Diastolic BP | -1.67 (-3.82, 0.47) | 0.13 | -1.63 (-3.95, 0.68) | 0.17 |  |
| HDL-c | -0.71 (-2.88, 1.47) | 0.52 | -0.90 (-3.74, 1.94) | 0.53 |  |
| Triglycerides | 1.11 (-11.4, 13.6) | 0.86 | 4.29 (-6.58, 15.2) | 0.44 |  |
| LDL-c | -1.74 (-9.17, 5.68) | 0.64 | 5.01 (-2.14, 12.17) | 0.17 |  |
| BMI | 0.88 (0.044, 1.71) | 0.039 | -0.22 (-1.21, 0.78) | 0.67 |  |
| Waist Circumference | 1.81 (-0.30, 3.91) | 0.093 | -1.72 (-4.06, 0.63) | 0.15 |  |
| Mean Liver Fat Attenuation | 1.50 (-0.95, 3.94) | 0.23 | -0.54 (-2.87, 1.78) | 0.65 |  |
| HOMA-IR | 0.13 (-1.19, 1.44) | 0.85 | 0.38 (-1.50, 2.25) | 0.69 |  |
| Distal common carotid IMT | 0.0067 (-0.052, 0.065) | 0.82 | -0.018 (-0.065, 0.030) | 0.47 |  |
| Internal carotid IMT | 0.029 (-0.089, 0.15) | 0.63 | 0.031 (-0.065, 0.13) | 0.53 |  |
| Format: Odds Ratio [95% Confidence Interval]. IMT=intima-media thickness. CAC=coronary artery calcium.  *p-value of parameter estimate is <0.004 (Bonferroni corrected p-value threshold for significance)  1. Beta regression coefficients adjusted for age, BMI, education level, household income quartile, percent of life living in the U.S., exercise (in MET-min/week), alcohol consumption (yes/no), metabolic syndrome criteria (except for outcome of interest: HbA1c%, waist circumference, HDL, triglycerides, systolic BP), LDL, liver fat attenuation, and smoking status (only in males). | | | | | |
