# Supplementary material for: Inverse association between total bilirubin and type 2 diabetes in U.S. South Asian males but not females
Source: PLoS One. 2024 Feb 7;19(2):e0297685. doi: 10.1371/journal.pone.0297685 (PMC10849233; doi:10.1371/journal.pone.0297685)
Supplement: Attachment — Submitted filename: REVIEWERS COMMENTS plos.docx [file pone.0297685.s002.docx]

Visaria et al have investigated whether total bilirubin was inversely associated with T2D, MetS and imaging-based atherosclerosis measures in U.S South Asians, this is an important topic and a contributes to the understanding of blood glucose control, however there are minor and major concerns regarding this paper.

Minor.

1. This works lacks line numbers, making it difficult to refer
2. In the covariates, what do metabolic factors mean?
3. What does total exercise represent? Physical activity? How about other activities that are not exercises?
4. The primary outcome is not clear, both in the method and results section. Is it T2D or bilirubin?
5. Results section 3.3 can be best presented before

Major

1. Authors aimed to investigate the inverse association and not just association. With the few studies done in this area, which some have reported inverse association and others have reported no association; authors could investigate whether there is any association in the target population regardless of the direction of the association.
2. Authors have not shown clearly how they defined the outcome of interest, T2D. Show it clearly the results by FBG, 2-hr post OGTT or by HbA1c. The associations may be different and the explanations can differ too. In the current analysis, it is too general and it is difficult to understand the pathophysiology.
3. Please define metabolic syndrome clearly, and state which criteria has been used in your definition.
4. Revise your materials and methods section. Clearly show how data for the outcome and predictor variables were collected and analyzed.
5. Why did authors use linear regression analysis verifying approximate normality of continuous outcomes? Please use the appropriate methods to check for normality and decide what tests of association will suite your data.
6. Authors used both quartiles and tertiles in their analyses. It is not clear why they didn’t choose either of the two in participants’ characteristics and logistic regression analysis?
7. What is the clear role of sensitivity analyses in this observational study?
8. Authors have concluded that, they have found an inverse association between total bilirubin and T2D, but this was not the case in women, so it should be clearly stated.
