# Supplementary material for: Inverse association between total bilirubin and type 2 diabetes in U.S. South Asian males but not females
Source: PLoS One. 2024 Feb 7;19(2):e0297685. doi: 10.1371/journal.pone.0297685 (PMC10849233; doi:10.1371/journal.pone.0297685)
Supplement: Attachment — Submitted filename: Response to Reviewers_R2_PLOS One_MASALA.docx [file pone.0297685.s004.docx]

Response to Reviewers

PONE-D-23-00961_R1

December 9, 2023

Dear Dr. Fredirick Lazaro Mashili,

Thank you for the additional comments and suggestions regarding our study (ID: PONE-D-23-00961_R1) titled, “*Inverse Association between Total Bilirubin and Type 2 Diabetes in U.S. South Asian Males but not Females*”. We have addressed all concerns and believe the comments have improved our manuscript. Below are the corrections/responses to each of the remarks. Changes to the manuscript text are also marked as tracked changes in the manuscript file.

**Responses to Reviewer #3**

***1. Lines 99 - 101 "Given the high prevalence of unfavorable lipid profiles and visceral adiposity in U.S. SAs, both of which can affect or are driven by the liver, we hypothesized that bilirubin, also produced in the liver, may be associated with T2D in SAs. This assertion needs to be supported by reference.***

Thank you for this comment. We have now cited this statement (citation #22).

Revision: *“Given the high prevalence of unfavorable lipid profiles and visceral adiposity in U.S. SAs, both of which can affect or are driven by the liver [22], we hypothesized that bilirubin, also produced in the liver, may be associated with T2D in SAs.” (*Page 3, Lines 93-95)

**2. Line 266 and 268 "Invitro and in vitro experimental studies have shown that unconjugated bilirubin can act as a potent, lipid-soluble antioxidant and help prevent oxidative stress that can induce insulin resistance". This statement is not clear.**

Thank you for this clarification question. We have now reworded the sentence to clarify it and correct some typographical mistakes.

Revision: *“There are several plausible mechanisms for bilirubin’s protective association with diabetes. In-vitro and in-vivo experimental studies have shown that unconjugated bilirubin can act as a potent, lipid-soluble antioxidant and help prevent oxidative stress, which in turn may reduce insulin resistance [11].” (Page 7, Lines 256-258)*

**3. Line 283-287. The description of the role of testosterone in bilirubin metabolism also needs to be supported by the relevant citation.**

Thank you for this comment. We have now cited this statement with a seminal in-vitro study looking at testosterone’s role in bilirubin metabolism.

Revision: “*Testosterone has been shown to inhibit UDP- glucuronosyltransferase, decreasing bilirubin metabolism and potentially producing greater antioxidant and anti-inflammatory effects [37].” (Page 7, Lines 274-275)*

**4. To enhance the germane of the study. I suggest, a description of the clinical relevance of the study.**

Thank you for this comment. We have now added 2 sentences to put our findings into clinical context.

Revision: *“In conclusion, among SA adults in the United States, we found an inverse association between total bilirubin and T2D but not other cardiometabolic factors. This association was stronger in men than in women. Longitudinal studies among SAs are needed to uncover total bilirubin’s predictive or prognostic value in T2D and overall CV risk before it can be used clinically to improve CV risk stratification; nevertheless, our findings add to a growing number of studies in other populations purporting the role of bilirubin on CVD detection and prevention.”* (Page 8, Lines 314-317)

**Reviewer #4: Please counter check the study/analysis design on the methodology section of the abstract and the main manuscript. On the abstract it is written prospective analysis while on the main manuscript it says retrospective analysis. Please clarify and rectify that.**

Thank you for this comment. We conducted both a cross-sectional (retrospective) and prospective, exploratory analysis. Our main analysis is retrospective in nature but because we also had prospective data on our outcome of interest, we also explored the association between bilirubin and incident diabetes. This was designated to be exploratory analysis only due to the limited sample size. We removed the reference to ‘retrospective study’ in the Methods section and changed it to ‘secondary analysis’ to avoid confusion.

Revision: *“This was a secondary analysis of fully de-identified data.” (Methods, Page 3, Line 112)*

Sincerely,

Aayush Visaria
